# Validation of a digital tool for Moyers mixed dentition analysis: agreement and efficiency compared with the manual method

**DOI:** 10.1590/2177-6709.31.3.e262634.oar

**Published:** 2026-08-28

**Authors:** Mariana Vasconcellos Bazoli RODRIGUES, Luciana Rougemont SQUEFF, Amanda Cunha Regal de CASTRO, Antônio Carlos de Oliveira RUELLAS, Sergio Luiz MOTA-JÚNIOR

**Affiliations:** 1 Universidade Federal do Rio de Janeiro, Dental School, Department of Pediatric Dentistry and Orthodontics (Rio de Janeiro/RJ, Brazil).

**Keywords:** Mixed dentition, Orthodontics, Software, Clinical diagnosis, Dentição mista, Ortodontia, Software, Diagnóstico clínico

## Abstract

**Introduction:**

Mixed dentition analysis is essential for predicting space requirements and guiding early orthodontic decision-making. Moyers analysis is widely used due to its simplicity and clinical applicability, however, manual execution may increase execution time and introduce operator-dependent errors.

**Objective:**

This study aimed to develop and validate an automated digital tool based on Moyers analysis, to improve efficiency and support orthodontic education.

**Material and Methods:**

This cross-sectional study included 20 participants (10 undergraduate and 10 graduate dental students) who performed mixed dentition analysis on a standardized plaster model using the manual method and a software-based method at separate time points. Agreement between methods was evaluated using intraclass correlation coefficients (ICC), Bland-Altman analysis, and linear regression to assess proportional bias. Execution time was recorded and compared using paired t tests. Participant preference was also assessed.

**Results:**

Excellent reliability was observed (ICC > 0.95). No statistically significant differences were found between the manual and software-based methods (p > 0.05), and Bland-Altman analysis demonstrated substantial agreement without proportional bias. The software-based method reduced execution time by 38.2% (p < 0.001). All participants preferred the software-based method.

**Conclusion:**

The software-based method demonstrated agreement comparable to the manual Moyers analysis, while significantly reducing execution time. Its usability and efficiency support its application in orthodontic education and clinical workflow optimization.

## RESUMO

**Introdução:**

A análise da dentição mista é essencial para prever a necessidade de espaço e orientar a tomada de decisão no tratamento ortodôntico nessa fase. A análise de Moyers é amplamente utilizada devido à sua simplicidade e aplicabilidade clínica; entretanto, sua execução manual pode aumentar o tempo de realização e introduzir erros dependentes do operador.

**Objetivo:**

Este estudo teve como objetivo desenvolver e validar uma ferramenta digital automatizada baseada na análise de Moyers, para melhorar a eficiência e auxiliar o ensino em Ortodontia.

**Material e Métodos:**

Esse estudo transversal incluiu 20 participantes (10 estudantes de graduação e 10 de pós-graduação em Ortodontia), que realizaram a análise da dentição mista em um modelo de gesso padronizado utilizando o método manual e um método baseado em *software,* em momentos distintos. A concordância entre os métodos foi avaliada por meio do coeficiente de correlação intraclasse (ICC), análise de Bland-Altman e regressão linear, para avaliação de viés proporcional. O tempo de execução foi registrado e comparado utilizando-se o teste t pareado. A preferência dos participantes também foi avaliada.

**Resultados:**

Foi observada excelente confiabilidade (ICC > 0,95). Não foram encontradas diferenças estatisticamente significativas entre os métodos manual e baseado em *software* (p > 0,05), e a análise de Bland-Altman demonstrou concordância substancial sem viés proporcional. O método baseado em *software* reduziu o tempo de execução em 38,2% (p < 0,001). Todos os participantes preferiram o método baseado em *software*.

**Conclusão:**

O método baseado em *software* demonstrou concordância comparável à análise manual de Moyers, reduzindo significativamente o tempo de execução. Sua usabilidade e eficiência sustentam sua aplicação no ensino da Ortodontia e na otimização do fluxo de trabalho clínico.

## INTRODUCTION

Mixed dentition is the developmental stage during which deciduous and permanent teeth coexist in the oral cavity, typically occurring from early childhood to preadolescence.^1^ It can be divided into early and late phases, marked by the eruption of permanent molars and incisors, followed by the replacement of deciduous canines and molars by permanent canines and premolars.^1^ This period is critical for orthodontic diagnosis, as it enables assessment of arch space, prediction of unerupted permanent tooth position, and identification of discrepancies that may guide early intervention.^2^

Moyers analysis is widely used in mixed dentition to predict the mesiodistal width of unerupted canines and premolars, based on the sum of the mandibular incisors, which are selected due to their early eruption, measurement reliability, and strong correlation with other tooth groups.^1^ Developed at the University of Michigan from a Northern European white sample, the method uses probability tables ranging from 5% to 95%, with the 75^th^ percentile being commonly recommended. Its clinical applicability is enhanced by its simplicity, rapid use, and the absence of radiographic requirements.^3^

In this context, the present study aimed to develop and validate an automated digital tool based on Moyers analysis, to improve diagnostic efficiency and facilitate student learning. Process automation may reduce measurement variability and optimize execution time, with potential applications in both educational and clinical settings. The null hypotheses tested were: (1) no difference between measurements obtained using the conventional method and the digital tool; and (2) no difference in the time required to perform the analysis between methods.

## MATERIAL AND METHODS

This cross-sectional study was approved by the Research Ethics Committee of Universidade Federal do Rio de Janeiro (UFRJ) (CAAE no. 96735926.3.0000.0268).

**Figure 1:**
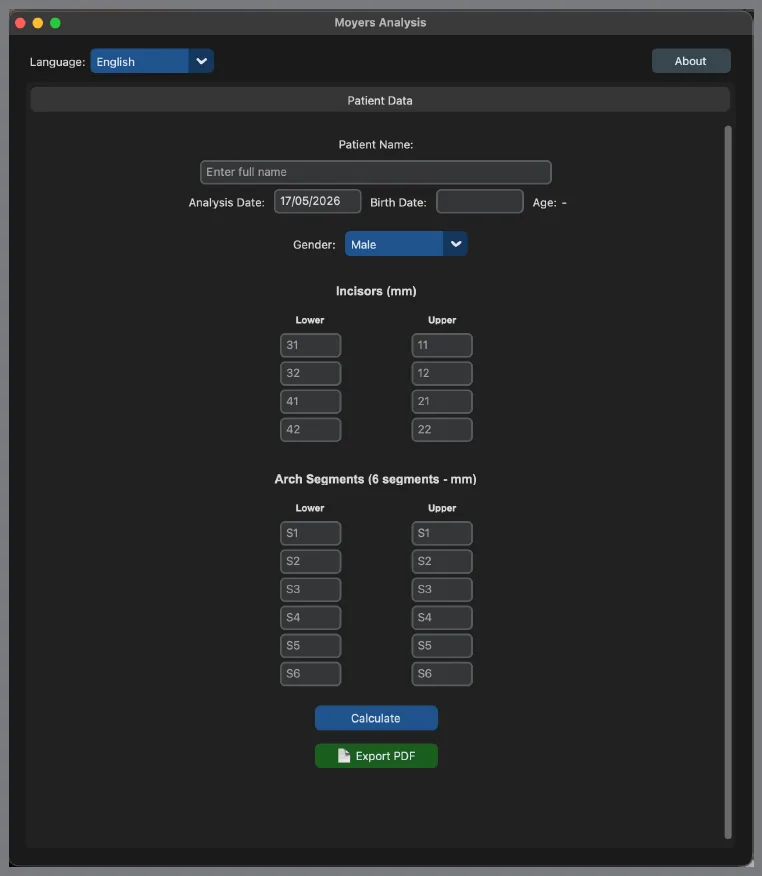
Interface of the developed software. After completing the required fields, the user selects “Calculate” to obtain the mixed dentition arch length discrepancy. Selecting “Export PDF” generates a report containing patient information and analysis results. The user can select the interface language: English, Spanish, Chinese, or Portuguese.

Initially, an open-access software was developed in Python by the authors, to automate the calculations required for mixed dentition analysis (Fig. 1). The software is available for download at the following link:

*doi:* *https://drive.google.com/drive/folders/1mAOXbK0IsGi7eszjexFdysu-tW_0HN8H?usp=share_link*

And it is compatible with Windows and macOS platforms. To verify computational accuracy, all values from the Moyers probability tables were tested at the 75^th^ percentile, as recommended, and complete agreement was observed, confirming that the software performed as intended.

A statistical power analysis was performed based on a previous study.^4^ A minimum sample of 20 individuals was required to achieve an 85% probability of obtaining 95% confidence intervals with margins of error smaller than 0.3 mm for the measurements analyzed.

The sample consisted of 20 participants, including 10 undergraduate dental students and 10 graduate orthodontic students. All participants were regularly enrolled and had prior knowledge of mixed dentition analysis using the Moyers method. Inclusion criteria were: voluntary agreement to participate, current enrollment in an undergraduate or graduate dental program at UFRJ, and current or previous completion of orthodontics coursework. Exclusion criteria included self-reported difficulty performing mixed dentition analysis or difficulty using computers. All participants provided written informed consent prior to participation.

All participants performed the mixed dentition analysis using the same previously selected orthodontic plaster model obtained from the archives of the UFRJ Graduate Program in Orthodontics. The model represented the mixed dentition stage, with retained deciduous canines and molars, and erupted permanent incisors and first molars. This standardization enabled direct comparison between methods and among participants, by eliminating variability associated with different dental models or arch characteristics.

Each participant performed mixed dentition analysis using two different methods at separate time points. At the first session (T_1_), the conventional method was used. Measurements were obtained from the plaster model using a bow divider (ICE^®^, São Paulo/SP, Brazil) and a millimeter ruler (Maquira, Maringá/PR, Brazil), followed by manual calculations recorded on a standardized form with reference to the Moyers probability tables at the 75^th^ percentile. Predicted values for the maxillary and mandibular arches were documented. To assess method error, the examiners repeated the measurements after a 30-day interval.

After a 30-day interval, the same participants repeated the analysis on the same plaster model using the software developed by the investigators for Moyers analysis (T2). During this session, participants used a notebook computer (Apple^®^, Cupertino, CA, USA) with the software preinstalled. A bow divider (ICE^®^, São Paulo, SP, Brazil) and a millimeter ruler (Maquira, Maringá/PR, Brazil) were also available for measurements, and paper and a ballpoint pen were provided for auxiliary notes if needed. Method error was assessed by repeating the measurements after an additional 30-day interval.

The time required for each operator to complete the analysis at T1 and T2 was recorded. Participants were not informed that timing was being performed, in order to minimize measurement bias. Time differences between methods were compared using paired t tests.

After completing the T2 measurements, participants were asked to indicate their preferred method by answering the following question: *“Which method did you prefer to use? ( ) Conventional; ( ) Computer-based software”*.

In the comparative analysis, no statistically significant differences were observed between T1 and T2 measurements for either undergraduate or graduate students, indicating adequate inter-observer reproducibility. In addition, intraclass correlation coefficients (ICCs) demonstrated excellent reliability, with values exceeding 0.95 for both groups. Based on these findings, participants were pooled into a single sample (n = 20), and subsequent analyses were conducted according to the method used (manual vs software-based). Analyses were performed separately for the maxillary and mandibular arches, because discrepancy values differ between arches and should not be combined in a single analysis.

Statistical analysis was performed using SPSS software (version 24; IBM Corp, Armonk, NY, USA). All measurements were repeated after a 30-day interval, to assess intra-observer reproducibility (ICCs).

One-sample t tests were used to evaluate whether discrepancies existed between paired measurement sets, with the test value set at zero. No statistically significant differences were observed in these comparisons. Therefore, the two measurements obtained from each participant were averaged, to represent the manual and software-based methods for subsequent analysis. Bland-Altman^5^ analysis was performed using Microsoft Excel (version 16 for macOS; Microsoft Corp, Redmond, WA, USA) and SPSS software, and Bland-Altman plots were generated to assess agreement between the manual and software-based methods. This analysis provided a visual assessment of agreement in arch length discrepancy values between methods. In addition, linear regression analyses were conducted to evaluate the presence of proportional bias. Statistical significance was set at p < 0.05.

## RESULTS

Data distribution was assessed using the Shapiro-Wilk test and demonstrated normality (p > 0.05), allowing the use of parametric tests for both discrepancy values and analysis time.

**Figure 2:**
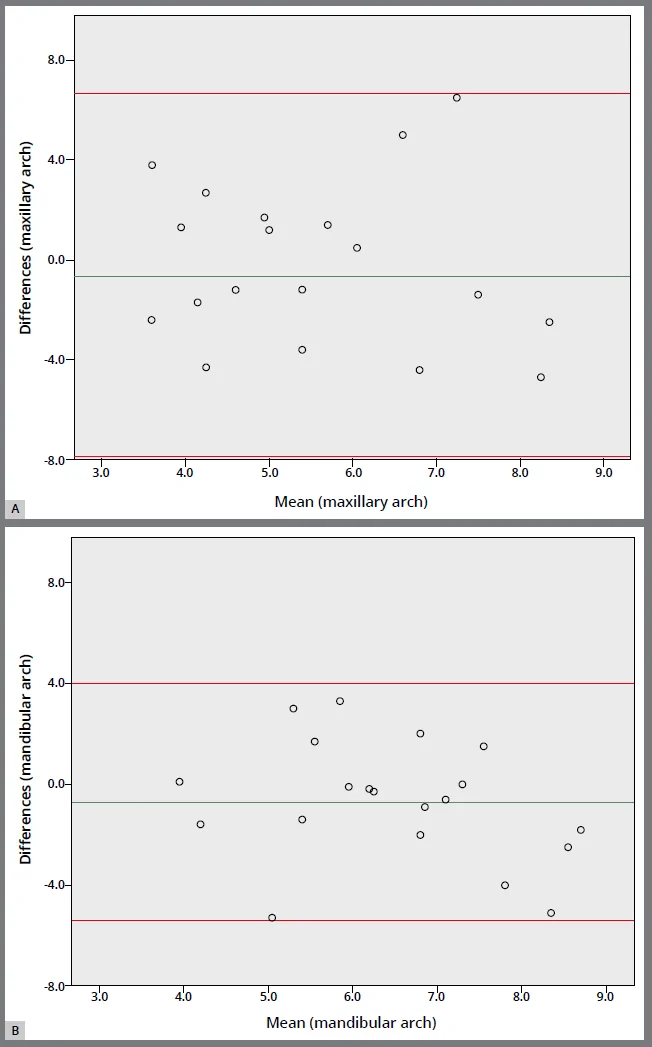
Bland-Altman plots illustrating agreement between manual and software-based measurements for the maxillary (**A**) and mandibular (**B**) arches.

Repeatability of the paired measurements demonstrated excellent reliability, with ICCs exceeding 0.95. Table 1 presents the mean biases, standard deviations, confidence intervals, and p-values for the paired comparisons between methods. One-sample t tests revealed no significant differences between paired methods, indicating substantial agreement. Figure 2 shows the Bland-Altman plots for method comparisons. Overall, Bland-Altman analysis indicated no fixed bias between the evaluated methods. The mean difference between the manual and software-based methods was -0.61 mm for the maxillary arch and -0.71 mm for the mandibular arch.

**Table 1:** Mean biases, standard deviations ( SD ), confidence intervals ( CI ), and p-values for method comparisons.

	Mean bias	SD	95% CI	p
Manual - Software (maxillary)	-0.61	3.71	-7.88 to 6.66	0.434
Manual - Software (mandibular)	-0.71	2.39	-5.39 to 3.97	0.221

In each plot in Figure 2, the central line represents the mean difference between the evaluated methods. Each point represents the difference between paired measurements obtained with the two calculation methods. In all plots, the central line was close to 0.00, indicating agreement between methods. The upper and lower lines represent the 95% limits of agreement. Values within these limits indicate substantial agreement between methods.

The results of the linear regression analyses are presented in Table 2. None of the comparisons showed statistically significant t scores, indicating the absence of proportional bias. Accordingly, the null hypothesis was accepted, demonstrating no systematic tendency for differences to increase or decrease, relative to the mean bias illustrated in the Bland-Altman plots.

**Table 2:** Summary of the linear regression analysis.

Arch	Method	t	P
Maxillary	Manual - Software	-1.26	0.222
Mandibular	Manual - Software	-1.27	0.218

Table 3 presents the time required for each measurement method. The software-based method was 38.2% faster than the conventional method.

**Table 3:** Time required by participants to perform arch length discrepancy analysis using each method: mean time (Time), minimum (Min), maximum (Max), standard deviation (SD), and p-value (P).

Method	Time, minutes	Min	Max	SD	p
Manual	18.6	11.2	26.7	3.8	<0.001*
Software	11.5	7.7	18.8	2.9	

* p<0.05, paired t test.

When asked which method they preferred to use, all participants indicated the software-based method.

## DISCUSSION

Mixed dentition analysis represents a fundamental component of orthodontic diagnosis, as it enables prediction of the space required for the eruption of permanent canines and premolars, and supports early clinical decision-making. Among the available methods, Moyers analysis remains widely used due to its simplicity, clinical applicability, and lack of radiographic requirements.^1^ However, manual execution requires repeated consultation of probability tables and arithmetic calculations, which may increase execution time and introduce errors, particularly in academic settings or high-demand clinical environments.^3^

Although straightforward and effective, the traditional analysis involves repeated consultation of probability tables and manual calculations, which may lead to uncertainty, delays, and an increased risk of human error, particularly in high-demand clinical environments or among students in the learning phase.^6^ With ongoing technological advances and the growing integration of digital tools into dental practice, opportunities have emerged to develop software that automates such analyses, improving efficiency, accuracy, and accessibility.^7^ Accordingly, the present study aimed to develop a digital tool that automates Moyers analysis, enabling students and clinicians to input measured values and obtain immediate estimates of required space and arch length discrepancy without manual calculations.

In the present study, comparison between the conventional method and the proposed digital tool demonstrated adequate agreement between measurements, as evidenced by Bland-Altman analysis. Most values remained within the 95% limits of agreement for both the maxillary and mandibular arches, indicating that the digital tool produced measurements comparable to those obtained with the conventional method. Linear regression analysis further showed no proportional bias (p > 0.05), indicating that differences between methods did not vary across the measurement range. Although some points demonstrated differences approaching or exceeding 2 mm, this level of dispersion is consistent with variability reported in orthodontic measurement studies and may be influenced by operator-related measurement variability and inherent limitations of mixed dentition prediction methods. Unlike investigations based on single linear measurements, mixed dentition analysis involves multiple sequential steps, including tooth width measurement, which may contribute to cumulative measurement variability between methods. Minor variations between methods have been reported to fall within clinically acceptable ranges, typically around 1 to 1.5 mm, with occasional differences approaching 3 mm, depending on measurement conditions.^8^ ^,^ ^9^ These findings are consistent with previous studies evaluating digital and conventional measurement techniques, which have reported high agreement and minimal mean differences between methods (approximately -0.01 to -0.08 mm).^10^

Regarding execution time, the software-based method demonstrated significantly superior performance, compared with the conventional method, with a mean reduction of 38.2% in the time required to complete the analysis (p < 0.001). This finding is consistent with previous studies showing that digitizing manual steps in orthodontic diagnosis enhances efficiency without compromising measurement quality.^11^ In high-demand clinical settings and academic environments, this reduction in execution time may represent a meaningful benefit, by improving productivity and optimizing clinical and instructional time.

The absence of statistically significant differences between undergraduate and graduate students in the manual method suggests that minimal prior knowledge of Moyers analysis was sufficient for procedure execution, regardless of training level. This finding supported pooling the participants for analysis and highlights the potential of the digital tool as an educational resource, capable of assisting both students in training and experienced clinicians. Previous studies have emphasized that digital tools applied to orthodontic education can contribute to standardization of analyses and reduction of operational errors during academic training.^7^

In addition, questionnaire responses indicated a favorable perception of the digital tool among participants. These qualitative findings are consistent with studies evaluating the acceptance of software and digital applications in dental practice, which emphasize usability and reduced operational complexity as key factors influencing adoption in clinical and academic settings.^11^

Beyond the validation of the proposed digital tool, the literature indicates that classic mixed dentition analyses may present limitations when applied to populations different from those used in their original formulation.^12^ ^-^ ^15^ Although the present study did not aim to propose new population-specific equations, automating Moyers analysis through a digital tool represents a relevant advancement, by reducing operational errors and standardizing the calculation process. Moreover, such a platform may facilitate future adaptations or the incorporation of population-specific predictive models.^16^

Despite the promising results, some limitations should be considered. The use of a single standardized dental model, although allowing control of variability, limits extrapolation of the findings to different malocclusion patterns and arch configurations. Future studies including more diverse samples, multiple dental models, and direct clinical application are necessary to further validate the proposed tool.^17^ ^-^ ^20^

Based on the present findings, the developed digital tool demonstrated adequate agreement with the manual Moyers method, while significantly reducing execution time, supporting its use as a reliable and efficient alternative for mixed dentition analysis. Automation of this process may contribute to optimization of orthodontic diagnosis and modernization of teaching and clinical practice without compromising measurement reliability.

## CONCLUSION

No statistically significant differences were observed between measurements obtained by undergraduate and graduate students or between the manual method and the digital tool in mixed dentition analysis using the Moyers method. However, the software-based method required significantly less execution time and was preferred by participants, indicating its potential application in orthodontic education.

## Data Availability

All data generated or analyzed during this study are included in this published article.
